# Association of Time to Antibiotics With Outcome in Pediatric Patients Receiving Chemotherapy for Cancer With Fever in Neutropenia—An International Individual Patient Data Meta‐Analysis

**DOI:** 10.1002/cam4.71512

**Published:** 2026-01-11

**Authors:** Amelie L. Salomon, Roland A. Ammann, Catherine Aftandilian, Konrad Bochennek, Eva Brack, Lee Dupuis, Caitlin W. Elgarten, Adam Esbenshade, Gabrielle M. Haeusler, Mia Karamatsu, Mette B. Moenster, Bob Phillips, Emily Schaeffer, Lillian Sung, Athanasios Tragiannidis, Nadja H. Vissing, Christa Koenig

**Affiliations:** ^1^ Pediatric Hematology/Oncology, Department of Pediatrics, Inselspital, Bern University Hospital University of Bern Bern Switzerland; ^2^ StatConsult Ammann Burgdorf Switzerland; ^3^ Faculty of Medicine University of Bern Bern Switzerland; ^4^ Pediatric Hematology/Oncology Stanford University Palo Alto California USA; ^5^ Pediatric Hematology and Oncology, Hospital for Children and Adolescents Johann Wolfgang Goethe University Frankfurt Germany; ^6^ Division of Haematology/Oncology The Hospital for Sick Children Toronto Ontario Canada; ^7^ Division of Oncology Children's Hospital of Philadelphia Philadelphia Pennsylvania USA; ^8^ Department of Pediatrics Vanderbilt University Medical Center and the Monroe Carell Jr. Children's Hospital at Vanderbilt Nashville Tennessee USA; ^9^ Department of Infectious Diseases Peter MacCallum Cancer Centre Melbourne Victoria Australia; ^10^ Department of Infection Diseases Royal Children's Hospital Parkville Victoria Australia; ^11^ Pediatric Emergency Medicine Stanford University Palo Alto California USA; ^12^ Department of Pediatrics and Adolescent Medicine Copenhagen University Hospital, Rigshospitalet Copenhagen Denmark; ^13^ Centre for Reviews and Dissemination University of York York UK; ^14^ Department of Paediatric Haematology and Oncology Leeds Children's Hospital Leeds UK; ^15^ 2nd Paediatric Department, Faculty of Health Sciences Aristotle University of Thessaloniki, AHEPA Hospital Thessaloniki Greece

**Keywords:** antibiotic administration, febrile neutropenia, fever in neutropenia, pediatric oncology, time to antibiotics

## Abstract

**Purpose:**

Fever in neutropenia (FN) is a potentially lethal complication of chemotherapy for cancer. Prompt administration of broad‐spectrum antibiotics is standard of care. Despite conflicting results on the association of time to antibiotics (TTA) with outcomes, TTA limits are used as FN quality measure both in adult and pediatric oncology. This individual patient data (IPD) meta‐analysis studied the association between TTA and outcomes in pediatric patients with FN.

**Patients and Methods:**

IPD on TTA in pediatric patients with FN receiving chemotherapy for any malignancy was collected internationally. Three‐level mixed binomial logistic regression analyzed the association of TTA with safety relevant events (SRE; death, admission to intensive care unit [ICU], bacteremia), primarily in patients with severe disease at presentation and secondarily in all patients.

**Results:**

Data on 4006 FN episodes in 2073 patients, diagnosed 2016–2023, were reported from 15 study sites in eight countries. Median TTA was 61 min overall and 53 min in the 345 (8.6%) episodes with severe disease at presentation. Among these with severe disease, an SRE was reported in 119 (34%) episodes. Longer TTA (> 60 vs. ≤ 60 min) was associated with less SRE (odds ratio, 0.41; 95% CI, 0.24–0.70). This primary finding was confirmed in secondary and additional exploratory analyses.

**Conclusion:**

This large, international and adequately powered IPD meta‐analysis found no association between shorter TTA and improved clinical outcomes in pediatric patients with FN. This finding was consistent across analyses. These results challenge the continued use of TTA limits as a quality measure for pediatric oncology centers.

## Graphical abstract

In an analysis of 4006 FN episodes from 2073 patients across 15 study sites in eight countries, no association was observed between shorter time to antibiotics (TTA) and reduced incidence of safety‐relevant events—including death, ICU admission, or bacteremia—regardless of whether patients presented with or without severe disease.

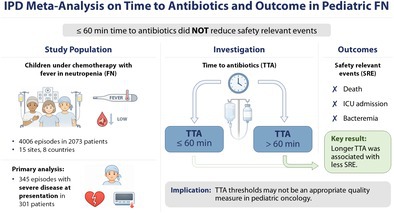

## 1 Introduction

Fever in neutropenia (FN) is the most common emergency in children undergoing chemotherapy for cancer and a leading cause for hospital admission. When the absolute neutrophil count falls below 0.5 G/L the risk of life‐threatening bacterial infection increases [1]. Consequently, patients and caregivers are advised to seek immediate medical evaluation in case of fever, and the standard of care mandates the prompt administration of broad‐spectrum antibiotics [2, 3, 4]. In some certification processes for pediatric oncology centers, for example, by the German Cancer Society, time to antibiotic administration (TTA) is utilized as a quality of care metric [5]. As a result, substantial resources have been allocated by various centers to minimize TTA [6, 7, 8, 9, 10, 11, 12]. Despite these established practices, the impact of TTA on patient outcomes remains unclear [13, 14]. Recent studies have not demonstrated a clear association between TTA and patient outcomes, leading to discussions regarding the appropriateness of TTA as a quality of care measure [15, 16, 17].

Emerging evidence suggests that FN patients may be stratified into two groups based on clinical presentation [14, 16, 17]. First, patients with severe disease at FN presentation may benefit from shorter TTAs, which supports recommendations to aim at TTAs ≤ 60 min, or even shorter intervals. Second, patients who are clinically stable at FN presentation may not benefit from expeditious antibiotic administration, rather allowing time to complete investigations and a period of observation [14, 16, 17].

A major challenge in analyzing the relationship between TTA and FN outcomes is the presence of triage bias [13]. Triage bias refers to the fact that patients presenting with more severe disease usually receive antibiotics more rapidly. This leads to the counterintuitive finding that a shorter TTA is associated with worse outcomes in unadjusted analyses [13, 15, 16]. To account for this bias, analytical approaches incorporating covariates, covariate scores, or stratified analyses based on disease severity at presentation are necessary, along with large sample sizes to ensure adequate statistical power.

This study aimed to use individual patient data (IPD) to perform a meta‐analysis on the association between TTA with outcomes in children presenting with FN while undergoing chemotherapy for cancer, primarily in patients with severe disease at FN presentation and secondarily in all patients.

## 2 Methods

### 2.1 Study Design, FN Episodes and Patients

The members of the Umbrella research group, an international expert panel to conquer infectious challenges in children with cancer [18], representing 19 pediatric oncology centers from four continents, were invited in November 2022 to contribute data on FN episodes from January 01, 2016 to December 31, 2023. Data was collected from 15 tertiary centers in eight high‐income countries in three continents, that is, Australia, Europe and North America. All centers had access to the intensive care unit (ICU). Data had been collected and stored electronically in these centers either for routine clinical use, for use in data quality measures like certifications, or within pro‐ or retrospective clinical studies. Data was extracted electronically (manually in one center) from patients' charts, from quality measure databases, or from clinical study databases, respectively.

Inclusion criteria were age younger than 18 years at FN presentation, diagnosis of any malignancy, and treatment with myelosuppressive chemotherapy. Therefore, this dataset represents a blend of newly collected data and a pooled retrospective cohort, combining IPD from different sources to enable comprehensive analysis. Inclusion criteria for study sites were the ability to provide a minimum set of mandatory variables per patient and per FN episode, and the respective definitions locally used for the variables reported (Table S1).

### 2.2 Objectives

The primary objective was to investigate the association between TTA and safety relevant events (SRE) in children and adolescents undergoing chemotherapy for cancer with severe disease at FN presentation. Severe disease at FN presentation was defined as severe sepsis or reduced clinical condition (i.e., a general deterioration in physical health) at presentation with FN, assessed at first contact by the responsible physician (see Table S1, for definition by center).

Secondary objectives were to investigate the association between TTA and SRE in (A) all pediatric patients undergoing chemotherapy for cancer presenting with FN regardless of severity at presentation, (B) according to the patients' location at fever detection, (C) based on an alternatively defined TTA, that is, time from fever detection to start of antibiotics, and (D) to investigate the association between TTA and the SRE composites, that is, serious medical complication (SMC) and bacteremia, as defined below.

### 2.3 Definitions

#### 2.3.1 Procedures and FN Definition

The study collected data on FN episodes occurring in in‐ and outpatients. Inpatients were either hospitalized or developed a fever during planned hospital visits. Outpatients developed fever outside the hospital, for example, at home. Neutropenia was defined as an absolute neutrophil count < 0.5 G/L or < 1 G/L and expected to decline to < 0.5 G/L within 48 h [19]. Fever was defined according to the local center (Table S1). The most frequent definitions were ≥ 38.5°C or ≥ 39.0°C once or ≥ 38.0°C twice within 1 h [19, 20].

#### 2.3.2 Time

For most analyses, TTA was defined as time span between arrival at the hospital (or time of fever detection, respectively, for inpatients) and start of antibiotics. If no exact timespan was known, sites had to minimally provide information on whether TTA was up to or longer than 60 min. For secondary analysis (C), an alternative TTA was defined as time from fever detection to start of antibiotics.

#### 2.3.3 Severe Disease at FN Presentation

Severe disease at FN presentation was defined as severe sepsis (according to slightly differing local definitions, including fulfillment of Goldstein et al. definitions [21], administration of fluid bolus for hemodynamic instability, or initiation of vasopressor therapy) or reduced clinical condition (i.e., a general deterioration in physical health) or assignment to a high triage category at presentation with FN. Severity was assessed at first contact by the responsible physician. Details on center‐specific definitions are provided in Table S1.

#### 2.3.4 Outcomes

Outcomes were assessed per FN episode. The primary outcome was the occurrence of any SRE, defined as bacteremia and/or SMC. Bacteremia was defined as the detection of a recognized pathogen from at least one blood culture [22]. This includes viridans group streptococci in the setting of concomitant mucosal barrier injury. A common commensal identified in a single blood specimen is considered a contaminant [22]. An SMC was defined according to a modified consensus definition [19] as death due to any cause during FN or admission to ICU, high dependency unit or other critical care unit for organ support, that is, not for monitoring only. The modification here consists of leaving out severe sepsis as an SMC defining event due to multiple reasons: (1) relevant discrepancy between the current research definition of septic shock and the clinical use of this term, (2) the further increase of this discrepancy in the setting of chemotherapy and FN because both fever and neutropenia are components of the definition of septic shock. The same is true for other direct effects of chemotherapies frequently used in pediatric oncology, like increased liver enzymes, and disturbed coagulation parameters [21], (3) the definitions used clinically for severe sepsis vary from institution to institution, reflecting the missing clinical usefulness of the research criteria.

### 2.4 Data Processing and Quality Assurance

**FIGURE 1:**
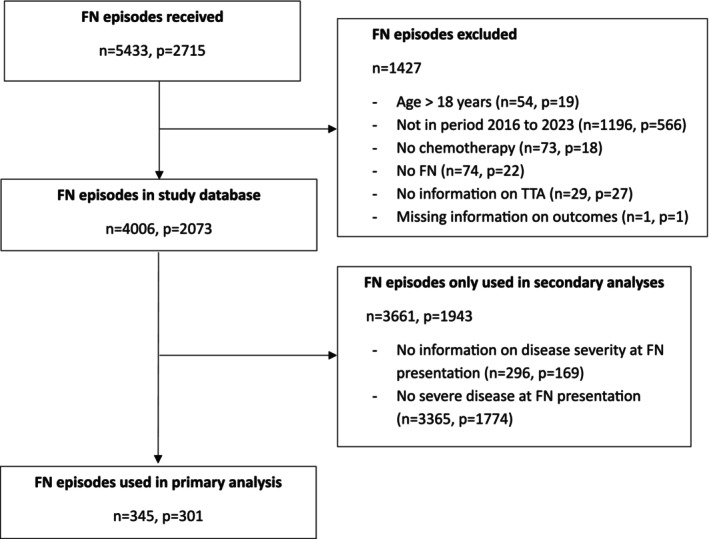
Flowchart of patients and episodes analyzed.

All data was received as .csv or .xlsx files which were imported into the R software [23]. Quality checks was performed by the lead site. Implausible data were confirmed with the contributing site. Figure 1 displays aggregated study‐wide information on episodes and patients included.

### 2.5 Statistical Analyses

The statistical analysis plan, including a power analysis for the primary objective and outcome, was defined in the study protocol prior to the analysis. Descriptive statistics using standard methods were used. Missing data were not imputed and complete case analysis was performed.

For the primary objective, two predefined analyses of the association between TTA and SRE were performed in the subset of patients with severe disease at FN presentation. First, TTA was analyzed as a binary variable (≤ 60 min vs. > 60 min), that is, binomial logistic three‐level mixed regression of SRE on this binary TTA, with random intercepts per patient, nested within country, was done to account for multiple episodes per patient. For this analysis on binary TTA, a power analysis had been made using the “power.prop.test” function from the “pwr” library in R [24], based on the proportions of 44% (four of nine) FN with SRE at TTA ≤ 60 min versus 60% (16 of 27) with SRE at TTA > 60 min reported elsewhere [14]. This power analysis led to a sample size of 2 × 165 = 330 episodes of FN with severe disease at presentation needed to reach a power of 90% to detect an increase of proportions from 44% to 60% at a one‐sided alpha of 0.05, corresponding to a power of 83% to detect such a difference in proportions at a two‐sided alpha of 0.05.

Second, if precise TTA was available, logistic three‐level mixed regression of SRE on categorized TTA was done, again with random intercepts per patient, nested within country. For categorization of TTA, the adjacent categories method was used to identify time intervals with significantly different risk of SRE. At start of this analysis, TTA was divided into 17 categories (eight intervals of 15 min up to 120 min, and eight 30 min intervals up to 360 min). Then adjacent time categories with the least significant differences were combined step by step, until only categories with at least tendentially (*p* ≤ 0.2) risk of SRE remained. This resulted in three categories, ≤ 30 min, 31–45 min and > 45 min.

Unexpectedly, our primary clinical hypothesis that a longer TTA would be associated with worse outcome in these high risk FN episodes was not confirmed in these analyses. To further challenge this result, four exploratory analyses not predefined in the study protocol were done. The first exploratory analysis accounted for the unexpectedly high heterogeneity of proportions of episodes with severe disease at FN presentation between datasets: the inverted proportion of FN episodes with severe disease at presentation was calculated and then tested for a direct association with SRE and for an interaction on the association of TTA with SRE. Proportions were defined per dataset, which was mostly equivalent to country, so for these analyses two‐level instead of three‐level mixed logistic regression, with random intercepts only per patient, was used. The second exploratory analysis introduced a propensity score balancing the probability of treatment assignment with observed baseline variables developed in an earlier study on TTA [14]: this score was calculated as continuous variable for each episode. The third exploratory analysis performed a complete sweep of time limits defining short versus long TTA through the range of observed TTAs. The TTA time limit was thus not set statically at 60 min, like in the first analysis of the primary outcome described above, but was varied from 0 to 1200 min. The fourth exploratory analysis included location at fever detection and the interaction of TTA with this location as additional regression parameters.

For secondary objectives, analyses comparable to the primary analysis were performed.

Two‐sided tests were used for all analyses. Correspondingly, 95% confidence intervals (CI) were calculated.

R 4.4.1 was used for data manipulation and all analyses [23]. Specifically, the “glmer” function from the “lme4” library was used for mixed logistic regression [25].

### 2.6 Ethical Considerations

The cantonal ethics committee of Bern, Switzerland, approved the main study protocol for this IPD meta‐analysis (BASEC ID 2023‐00422) before the start of data collection and analysis (Text S1). Contributing study sites ensured that data had been collected and was shared according to the respective national and local regulations, including approval by further local ethics committees and informed consent where required. General consent for further use of clinical data was obtained from patients and/or their legal guardians or informed consent was waived in accordance with local regulations.

Data sharing agreements between the Department of Pediatrics, Inselspital, Bern University Hospital, Bern, Switzerland and the respective study sites were established where required. No personal or identifying data was collected.

## 3 Results

### 3.1 Patients and FN Episodes

In total, data on 5433 FN episodes from 2715 patients was submitted from 15 centers. Of these, 4006 FN episodes (74%) from 2073 patients (76%) could be included in the meta‐analysis (Figure 1). Of these 2073 patients, 888 (46%) patients were female, median age was 7 years (interquartile range [IQR], 3.5–12.5), and the most common diagnosis was acute lymphoblastic leukemia (850 patients, 43%; Table S2). Distributions of age, sex and diagnosis were comparable between study sites (Table S3) and episodes with ≤ 60 min versus > 60 min (Table S4). The median number of FN episodes per patient was 1 (IQR, 1–2; maximum, 11). Characteristics of FN episodes are displayed in Table S5.

### 3.2 Primary Outcome

The primary analysis was restricted to 345 (8.6%) FN episodes with severe disease at presentation, reported in 301 (15%) of 2073 patients from 13 sites in six countries in three continents. The site‐specific proportion of such episodes ranged from 2% to 43% (Table S3). Severe disease at FN presentation was assigned for severely reduced condition in 204 (59%) episodes, for severe sepsis in 124 (36%), and for high triage category in 114 (33%). Of the 301 patients, 125 (44%) patients were female, their median age was 9 (IQR 4–14), the most common type of malignancy was acute lymphoblastic leukemia (49%) (Table S2), and 38 (13%) patients had at least two FN episodes (median per patient 1; maximum 4).

**FIGURE 2:**
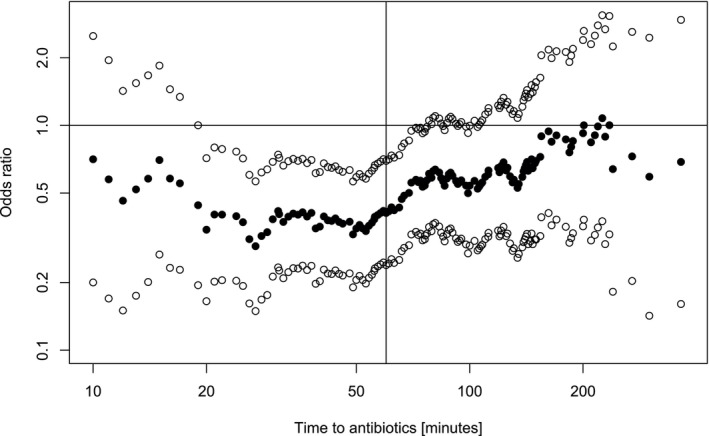
Association of time to antibiotics (TTA) and safety relevant events (SRE) sweeping the time limits defining short versus long TTA through the range of observed TTAs. Solid circles: Odds ratio. Open circles: 95% confidence interval.

An SRE was reported in 119 (34%) episodes with severe disease at FN presentation (9 deaths, 50 ICU admissions, 95 bacteremia). The median TTA was 53 min (IQR, 31–94; range, 0–1200), with shorter median TTA in episodes with SRE (40 min) versus without SRE (61 min). This was reflected in an odds ratio below unity (0.41; 95% CI, 0.24–0.70) to develop SRE in episodes with TTA > 60 min versus ≤ 60 min. The same association of longer TTA with lower SRE risk was seen in the categorical TTA analysis (Table 1). The four additional exploratory analyses, adjusting for the site‐specific proportion of episodes with severe disease at presentation, a published propensity score for SRE in FN [14], sweeping the limit to define short versus long TTA through the entire range of observed TTAs (Figure 2, showing odds ratio [solid circles] and 95% confidence interval [open circles]), and including location at fever detection and the interaction of TTA with this location as additional regression parameters, all demonstrated the same association (Figure 2; Table S6).

**TABLE 1:** Association between time from arrival at the hospital to start of antibiotics (TTA) and the occurrence of safety relevant events (SRE).

	All episodes		Results of three‐level mixed logistic regression		Episodes with severe disease at presentation		Results of three‐level mixed logistic regression		Episodes without severe disease at presentation		Results of three‐level mixed logistic regression	
	4006 FN episodes	692 SRE	Odds ratio (95% CI)	*p*	345 FN episodes	119 SRE	Odds ratio (95% CI)	*p*	3365 FN episodes	410 SRE	Odds ratio (95% CI)	*p*
Binary variable												
≤ 60 min	1996 (50%)	338 (49%)	1 (Reference)	—	191 (55%)	78 (66%)	1 (Reference)	—	1694 (50%)	204 (50%)	1 (Reference)	
> 60 min	2010 (50%)	354 (51%)	0.7 (0.57–0.84)	< 0.001	154 (45%)	41 (34%)	0.41 (0.24–0.70)	< 0.001	1671 (50%)	206 (50%)	0.75 (0.6–0.95)	0.015

	3998 FN episodes	691 SRE	Odds ratio (95% CI)	*p*	345 FN episodes	119 SRE	Odds ratio (95% CI)	*p*	3365 FN episodes	410 SRE	Odds ratio (95% CI)	*p*
Adjacent categories												
≤ 30 min	816 (20%)	162 (23%)	1 (Reference)	—	84 (24%)	42 (35%)	1 (Reference)	—	668 (20%)	84 (20%)	1 (Reference)	
31–45 min	640 (16%)	102 (15%)	0.84 (0.63–1.12)	0.24	68 (20%)	25 (21%)	0.67 (0.32–1.37)	0.270	548 (26%)	63 (15%)	0.87 (0.61–1.24)	0.45
> 45 min	2542 (64%)	427 (62%)	0.6 (0.48–0.76)	< 0.001	193 (56%)	52 (44%)	0.32 (0.17–0.59)	< 0.001	2149 (64%)	263 (64%)	0.71 (0.54–0.94)	0.016

Abbreviations: CI, confidence interval; FN, fever in neutropenia; SRE, safety relevant events; TTA, time to antibiotics.

### 3.3 Secondary Outcomes

In the entire set of FN episodes analyzable, median TTA was longer than in the subset of patients with severe disease at presentation (61 vs. 53 min), and episodes with SRE were less frequent (17% vs. 35%) (Table 1). Results of three‐level mixed logistic regression were comparable to the primary analysis (Table 1). Results were also comparable in analysis (A) of patients without severe disease at presentation (Table 1); in separate analyses according to the patients' location at fever detection (Table 2); analyzing time from fever detection to start of antibiotics (Table 3); and separate analyses of the SRE components, death and/or ICU admission, and bacteremia, as outcomes (Table 4).

**TABLE 2:** Association between time from arrival at the hospital to start of antibiotics (TTA) and the occurrence of safety relevant events (SRE) in inpatients versus patients not in study site at FN diagnosis, in patients with severe disease at FN diagnosis.

	Episodes in inpatients		Results of three‐level mixed logistic regression		Episodes not in study site		Results of three‐level mixed logistic regression	
	74 FN episodes	41 SRE	Odds ratio (95% CI)	*p*	206 FN episodes	64 SRE	Odds ratio (95% CI)	*p*
Binary variable								
≤ 60 min	44 (59%)	24 (59%)	1 (Reference)	—	122 (59%)	45 (70%)	1 (Reference)	—
> 60 min	30 (41%)	17 (41%)	1.11 (0.42–2.94)	0.83	84 (41%)	19 (30%)	0.38 (0.18–0.81)	0.012
Adjacent categories								
≤ 30 min	25 (34%)	13 (32%)	1 (Reference)	—	48 (23%)	24 (38%)	1 (Reference)	—
31–45 min	10 (14%)	7 (17%)	2.16 (0.44–10.57)	0.34	53 (26%)	15 (23%)	0.39 (0.65–0.95)	0.039
> 46 min	39 (52%)	21 (51%)	1.07 (0.39–2.98)	0.891	105 (51%)	25 (39%)	0.24 (0.09–0.6)	0.002

Abbreviations: CI, confidence interval; FN, fever in neutropenia; F‐TTA, time from fever to start of antibiotics; SRE, safety relevant events.

**TABLE 3:** Association between time from fever to start of antibiotics (FTA) and the occurrence of safety relevant events (SRE).

	All episodes		Results of three‐level mixed logistic regression		Episodes with severe disease at presentation		Results of three‐level mixed logistic regression		Episodes without severe disease at presentation		Results of three‐level mixed logistic regression	
	626 FN episodes	91 SRE	Odds ratio (95% CI)	*p*	95 FN episodes	32 SRE	Odds ratio (95% CI)	*p*	531 FN episodes	59 SRE	Odds ratio (95% CI)	*p*
Binary variable												
≤ 60 min	344 (55%)	43 (47%)	1 (Reference)	—	47 (49%)	14 (44%)	1 (Reference)	—	297 (56%)	25 (42%)	1 (Reference)	—
> 60 min	282 (45%)	48 (53%)	0.46 (0.21–1.04)	0.063	48 (51%)	18 (56%)	0.32 (0.07–1.43)	0.136	234 (44%)	34 (58%)	0.93 (0.22–3.99)	0.92
Adjacent categories												
≤ 30 min	326 (52%)	43 (47%)	1 (Reference)	—	43 (45%)	12 (38%)	1 (Reference)	—	283 (53%)	31 (53%)	1 (Reference)	—
31–45 min	9 (1%)	3 (3%)	1.54 (0.25–9.48)	0.64	2 (2%)	1 (3%)	0.35 (0.01–12.3)	0.56	7 (1%)	2 (3%)	63 (0.33–12,210)	0.12
> 45 min	291 (46%)	45 (49%)	0.49 (0.17–1.37)	0.17	50 (53%)	19 (59%)	0.23 (0.04–1.48)	0.12	241 (45%)	26 (44%)	1.04 (0.21–5.2)	0.96

Abbreviations: CI, confidence interval; FN, fever in neutropenia; F‐TTA, time from fever to start of antibiotics; SRE, safety relevant events.

**TABLE 4:** Association between time from arrival to start of antibiotics (TTA) and the occurrence of serious medical complications (SMC) and bacteremia.

	All patients		Results of three‐level mixed logistic regression		Patients with severe disease at presentation		Results of three‐level mixed logistic regression		Patients without severe disease at presentation		Results of three‐level mixed logistic regression	
	4006 FN episodes	182 SMC	Odds ratio (95% CI)	*p*	345 FN episodes	55 SMC	Odds ratio (95% CI)	*p*	3365 FN episodes	94 SMC	Odds ratio (95% CI)	*p*
Binary variable												
≤ 60 min	1996 (50%)	95 (52%)	1 (Reference)	—	191 (55%)	39 (71%)	1 (Reference)	—	1694 (50%)	40 (43%)	1 (Reference)	—
> 60 min	2010 (50%)	87 (48%)	0.59 (0.43–0.81)	0.001	154 (45%)	16 (29%)	0.37 (0.18–0.76)	0.006	1671 (50%)	54 (57%)	0.85 (0.52–1.38)	0.51
Adjacent categories	*n* = 3998											
≤ 30 min	816 (20%)	46 (25%)	1 (Reference)	—	84 (24%)	19 (35%)	1 (Reference)	—	668 (20%)	13 (14%)	1 (Reference)	—
31–45 min	640 (16%)	35 (19%)	1.0 (0.65–1.58)	0.97	68 (20%)	16 (29%)	0.99 (0.43–2.3)	0.98	548 (26%)	17 (18%)	1.53 (0.72–3.26)	0.27
> 46 min	2542 (64%)	101 (55%)	0.49 (0.34–0.71)	< 0.001	193 (56%)	20 (36%)	0.30 (0.13–0.67)	0.004	2149 (64%)	64 (68%)	0.99 (0.52–1.89)	0.98

	4006 FN episodes	601 Bacteremia	Odds ratio (95% CI)	*p*	345 FN episodes	95 Bacteremia	Odds ratio (95% CI)	*p*	3365 FN episodes	349 Bacteremia	Odds ratio (95% CI)	*p*
Binary variable												
≤ 60 min	1996 (50%)	293 (49%)	1 (Reference)	—	191 (55%)	61 (64%)	1 (Reference)	—	1694 (50%)	179 (51%)	1 (Reference)	—
> 60 min	2010 (50%)	308 (51%)	0.71 (0.58–0.87)	0.001	154 (45%)	34 (36%)	0.48 (0.28–0.83)	0.009	1671 (50%)	170 (49%)	0.74 (0.58–0.95)	0.017
Adjacent categories	*n* = 3998	*n* = 600										
≤ 30 min	816 (20%)	145 (24%)	1 (Reference)	—	84 (24%)	35 (37%)	1 (Reference)	—	668 (20%)	77 (22%)	1 (Reference)	—
31–45 min	640 (16%)	82 (14%)	0.75 (0.55–1.03)	0.075	68 (20%)	17 (18%)	0.54 (0.24–1.19)	0.12	548 (26%)	51 (15%)	0.76 (0.53–1.11)	0.16
> 46 min	2542 (64%)	373 (62%)	0.61 (0.48–0.77)	< 0.001	193 (56%)	43 (45%)	0.35 (0.19–0.66)	0.002	2149 (64%)	221 (63%)	0.68 (0.51–0.91)	0.01

Abbreviations: CI, confidence interval; FN, fever in neutropenia; SMC, serious medical complications; TTA, time to antibiotics.

## 4 Discussion

Both predefined analyses of the primary outcome showed that shorter TTA was associated with worse outcome, that is, a higher risk to develop safety‐relevant events, both bacteremia and serious medical complications. Four additional exploratory analyses were designed to challenge these counterintuitive results. These analyses, however, supported the initial analyses. Our results are in line with several smaller, single country studies in adult [26, 27, 28] and pediatric patients [14, 15, 16, 29, 30] who have not found an association of a longer TTA with worse outcome. This may be due to residual confounding or a wrong sense of security resulting from the focus on rapid antibiotic administration over thorough patient evaluation and the careful management of circulatory instability. The application of a sepsis screening tool with risk‐based assessment of each patient presenting with FN would be more relevant than uniform application of the isolated TTA metric. The results support that the participating centers have implemented FN guidelines into routine clinical practice that led to prompt recognition of children presenting with severe disease at FN presentation, leading to prompt initiation of antibiotic therapy. The secondary analyses in the unselected set of FN episodes, and using a different definition of TTA, also supported these results. We did not identify an alternative TTA cutoff associated with worse outcomes, using either the adjacent categories method or exploratory analyses sweeping the time threshold from 0 to 1200 min. Notably, 75% of episodes had a TTA shorter than 2 h, limiting the ability to assess the impact of longer TTAs on clinical outcomes.

The main strength of this analysis relies on the IPD design with a large international dataset. Automated data check and management pipelines were constructed, adapted to different data formats and units from the different sources for extensive quality checks and data format unification.

As expected in this multicenter study setting, there were differences in data quality, in definitions of FN itself and of outcomes, and lack of uniform modalities for temperature detection. Stringent eligibility criteria for participating centers, patients and episodes, together with site‐specific data pipelines including quality checks, were used to overcome this drawback. An additional exploratory analysis accounted for the unexpected high differences in the proportion of FN episodes reported with severe disease at presentation. The absence of data on time to neutrophil recovery represents an important limitation of this study, as the duration of neutropenia is a critical factor influencing the outcomes of FN.

In conclusion, this large, international and adequately powered IPD meta‐analysis, in pediatric patients undergoing chemotherapy for cancer presenting with FN to high‐resourced centers in high‐income countries, no association was observed between shorter TTA and improved clinical outcomes. This result was consistent across patients and episodes with and without severe disease at FN presentation. Results have to be validated outside the study setting and should not discourage aiming for fast assessment and treatment in pediatric FN, particularly in children with severe disease at presentation. Comparable to findings in adult oncology, these results do, however, clearly challenge the continued use of TTA limits as a quality measure for pediatric oncology centers and their corresponding emergency departments, be it in clinical practice or for certification purposes.

## Author Contributions

**Amelie L. Salomon:** data curation (equal), formal analysis (equal), writing – original draft (equal). **Roland A. Ammann:** conceptualization (equal), formal analysis (equal), methodology (equal), resources (equal), supervision (equal), visualization (lead), writing – original draft (equal), writing – review and editing (equal). **Catherine Aftandilian:** conceptualization (equal), data curation (equal), writing – review and editing (equal). **Konrad Bochennek:** conceptualization (equal), data curation (equal), writing – review and editing (equal). **Eva Brack:** conceptualization (equal), writing – review and editing (equal). **Lee Dupuis:** conceptualization (equal), writing – review and editing (equal). **Caitlin W. Elgarten:** conceptualization (equal), data curation (equal), writing – review and editing (equal). **Adam Esbenshade:** data curation (equal), writing – review and editing (equal). **Gabrielle M. Haeusler:** conceptualization (equal), data curation (equal), writing – review and editing (equal). **Mia Karamatsu:** data curation (equal), writing – review and editing (equal). **Mette B. Moenster:** data curation (equal), writing – review and editing (equal). **Bob Phillips:** conceptualization (equal), data curation (equal), writing – review and editing (equal). **Emily Schaeffer:** data curation (equal), writing – review and editing (equal). **Lillian Sung:** conceptualization (equal), data curation (equal), writing – review and editing (equal). **Athanasios Tragiannidis:** conceptualization (equal), data curation (equal), writing – review and editing (equal). **Nadja H. Vissing:** conceptualization (equal), data curation (equal), writing – review and editing (equal). **Christa Koenig:** conceptualization (equal), formal analysis (equal), funding acquisition (lead), methodology (equal), resources (lead), software (lead), supervision (equal), visualization (supporting), writing – original draft (equal).

## Funding

This trial was supported by a 2024/2025 Young Investigator Grant for patient‐oriented research, to C.K. from the Inselspital Bern, Switzerland. L.S. is supported by the Canada Research Chair in Pediatric Oncology Supportive Care. G.M.H. is supported by a National Health and Medical Research Investigator Grant.

## Conflicts of Interest

The authors declare no conflicts of interest.

## Supporting information


**Data S1:** cam471512‐sup‐0001‐DataS1.docx.

## Data Availability

Data from Switzerland are available for qualified researchers who wish to access the data following article publication; no end data Proposals should be directed to christa.koenig@insel.ch; to gain access, data requestors will need to sign a data access agreement. Data from other participating centers and countries have to be requested from the respective study center.
