# Supplementary material for: Association of Time to Antibiotics With Outcome in Pediatric Patients Receiving Chemotherapy for Cancer With Fever in Neutropenia—An International Individual Patient Data Meta‐Analysis
Source: Cancer Med. 2026 Jan 11;15(1):e71512. doi: 10.1002/cam4.71512 (PMC12790953; doi:10.1002/cam4.71512)
Supplement: Supplementary file 1 — Data S1: cam471512‐sup‐0001‐DataS1.docx. [file CAM4-15-e71512-s001.docx]

Data Supplement

Association of time to antibiotics with outcome in pediatric patients receiving chemotherapy for cancer with fever in neutropenia – an international individual patient data meta-analysis

Amelie L Salomon*, Roland A Ammann*, Catherine Aftandilian, Konrad Bochennek, Eva Brack, Lee Dupuis, Caitlin W Elgarten, Adam Esbenshade, Gabrielle M Haeusler, Mia Karamatsu^,^ Mette B Moenster, Bob Phillips, Emily Schaeffer, Lillian Sung, Athanasios Tragiannidis, Nadja H Vissing, Christa Koenig**

Content

**Table S1** Definitions used by study sites 2

**Table S2** Patient characteristics.* 4

**Table S3** Patient characteristics per study site 5

**Table S4** Patient characteristics stratified by TTA ≤ 60 minutes versus > 60 minutes.* 6

**Table S5** FN episode characteristics.* 7

**Table S6** Exploratory analyses: Association between time from arrival at the hospital to start of antibiotics (TTA) and the occurrence of safety relevant events (SRE) corrected for 1) the inverted proportion of FN episodes with severe disease at presentation, 2) a propensity score and 3) including location at fever detection and the interaction of TTA with this location as additional regression parameters 8

**Text S1** Study protocol 9

# 1

**Table S1** Definitions used by study sites.

| **Study center (Country)** | **Neutropenia** | **Fever limit** | **Death** | **Bacteremia** | **Reduced general condition at presentation** | **Severe sepsis at presentation** | **Triage categories used** | **Chemotherapy intensities** |
| --- | --- | --- | --- | --- | --- | --- | --- | --- |
| **Bern (Switzerland)** | <0.5 G/L, or <1  G/L and expected to decline to <0.5 G/L within 48h | ≥ 39.0°C once, or slightly elevated temperature (≥37.5°C) if the responsible physician decided to  treat as FN | Death due to any cause during FN | Detection of a recognized pathogen from one or mood blood cultures according to current definitions | More than “slightly reduced clinical condition” according to responsible physician | Definition adapted from Goldstein et al. fulfilled at presentation | 1-4, ≥3 implies severe disease at presentation | Expected neutropenia:  1 (none)  2 (≤10 days)  3 (>10 days)  4 (myeloablative) |
| **Copenhagen (Denmark)** | <0.5 G/L | >38.5°C or ≥38.0°C  that persists for >1 hour | Death due to any cause during FN | Detection of any bacterial pathogen from one or more blood  cultures | - | - | - | - |
| **Frankfurt (Germany)** | <0.5 G/L | ≥38.0twice within one hour, or  ≥38.5°C once | Death due to any cause during FN | Detection of a pathogen from one or more blood cultures according to  current definitions | More than “slightly reduced clinical condition” according to  responsible physician | Goldstein et al. fulfilled at presentation | 1-4, ≥3 implies severe disease at prsentation | - |
| **Melbourne (Australia)** | <1.0 G/l | ≥38.0 °C once | Death due to any case within 30 days of FN presentation | Detection of a recognized pathogen from one or more blood cultures. Common commensals (excluding oral viridians strep group) cultured from 2 or more blood cultures | Any of: GCS < 15; AVPU = V, P or U,  documented as 'severely unwell' (or equivalent) or severe sepsis or septic shock or blood pressure or respiration rate in MET criteria or fluid  bolus >40ml/kg | Goldstein et al. fulfilled at presentation | 1-5, ≤2 implies severe disease at presentation | 2 categories - chemotherapy more or less intensive than acute lymphoblastic leukemia maintenance style chemotherapy |
| **Philadelphia (United States)** | <0.2 G/L | ≥38.5 once or ≥38.0  °C 3 times within 24 hours, each measurement at least 2 hours apart | Death due to any cause during FN | Culture based methods only (did not include NGS) | Overlapping with sepsis | Hemodynamic instability requiring >20 ml/kg normal saline or initiation of vasopressors at presentation | - | - |
| **SPOG**  **(Switzerland)** | <0.5 G/L, or <1  G/L and expected to decline to <0.5 G/L within 48h | ≥38.5°C or ≥39.0°C  once, or slightly elevated temperature (≥38.0°C, ≥37.5°C  in patients repeatedly receiving  antipyretics) if the responsible | Death due to any cause during FN | Detection of a recognized pathogen from one or mood blood cultures according to current definitions | “Severely reduced general condition” according to responsible physician | Definition adapted from Goldstein et al. fulfilled within 6 hours from presentation | - | Expected neutropenia:  1 (none)  2 (≤10 days)  3 (>10 days)  4 (myeloablative) |

|  |  | physician decided to treat as FN |  |  |  |  |  |  |
| --- | --- | --- | --- | --- | --- | --- | --- | --- |
| **Stanford (United States)** | < 0.5 GI/l | ≥38.0 °C twice over one hour, or  ≥38.3°C once | Death due to any cause during FN | Detection of any bacterial pathogen from one or more blood cultures | - | Hemodynamic instability requiring > 20cc/kg NS or initiation of vasopressors at  presentation | Emergency Severity Index | - |
| **Thessaloniki (Greece)** | <0.5 G/L or expected to decline to <0.5 G/L within 48h | >38.3°C or ≥38.0°C  that persists for >1 hour | Death related to the underlying episode/infectio n | Positive blood culture with symptoms. In the case of coagulase negative Staphylococcus spp, two positive blood cultures were required. | Clinical condition (tachycardia, fever, hypotension, SIRS) | Goldstein et al. fulfilled at presentation | 1-3, 3 implies severe disease at presentation | 1. (none) 2. (<10 days) 3. (>10 days) 4. (myeloablative, FLAG, FLAG+ immunotherapy, high dose ARAC plus other chemo, HR ALL   blocks) |
| **Toronto (Canada)** | <0.5 G/L or expected to decline to <0.5 G/L within 48h | >38.3°C or ≥38.0°C  that persists for >1 hour | Death due to any cause during FN | Detection of any bacterial pathogen from one or more blood cultures | - | Use of inotropes (epinephrine, norepinephrine, dopamine, dobutamine, milrinone, and/or vasopressin)  within 4 hours | - | - |
| **UK (United Kingdom)** | <0.5 G/L | >38.0°C once | Death due to any cause during FN | Detection of a recognized pathogen from one or more blood cultures according to  current definitions | - | - | - | - |
| **Vanderbilt (United States)** | <0.5 G/l | >38.3°C or ≥38.0°C  that persists for >1 hour | Death due to any cause during FN | Detection of a recognized pathogen from one or more blood cultures. Common commensals (excluding alpha strept/Strept mitis) cultured from 2 or more  blood cultures | “Severely reduced general condition” according to responsible physician | Hypotension, chills | - | - |

Abbreviations: FN, fever in neutropenia; SIRS, Systemic Inflammatory Response Syndrome; SPOG, Swiss Pediatric Oncology Group; MET, medical emergency team; NGS, Next-Generation Sequencing;

| **Table S2** Patient characteristics.* |  | | | | |
| --- | --- | --- | --- | --- | --- |
|  | Total patients in |  | Patients with |  | Patients with |
|  | study |  | episodes with |  | episodes without |
|  |  |  | severe disease at |  | severe disease at |
|  |  |  | presentation |  | presentation |
| Number of patients | 2073 |  | 301 |  | 1774 |
| Age at FN diagnosis, years | n = 1620 |  | n = 207 |  | n = 1531 |
|  | 7 (3.5 to 12.5) |  | 9 (4 to 14) |  | 7 (3.5 to 12.5) |
| Sex | n = 1928 |  | n = 286 |  | n = 1704 |
| Female | 888 (46%) |  | 125 (44%) |  | 789 (46%) |
| Male | 1040 (54%) |  | 161 (56%) |  | 915 (54%) |
| Type of malignancy | n= 2002 |  | n = 286 |  | n = 1704 |
| Acute lymphoblastic leukemia | 850 (43%) |  | 141 (49%) |  | 707 (41%) |
| Acute myeloid leukemia | 156 (8%) |  | 16 (6%) |  | 118 (7%) |
| Hodgkin lymphoma | 68 (3%) |  | 8 (3%) |  | 59 (3%) |
| Non-Hodgkin lymphoma | 120 (6%) |  | 19 (7%) |  | 100 (6%) |
| Central nervous system tumor | 187 (10%) |  | 25 (9%) |  | 159 (9%) |
| Other solid tumors | 539 (27%) |  | 72 (25%) |  | 489 (29%) |
| Other malignancies | 82 (4%) |  | 5 (2%) |  | 72 (4%) |
| Relapsed malignancy | n = 1255 |  | n = 236 |  | 1146 |
| Yes | 142 (11%) |  | 35 (15%) |  | 126 (11%) |
| No | 1113 (89%) |  | 201 (85%) |  | 1020 (89%) |
| Continent |  |  |  |  |  |
| Australia | 380 (18%) | 76 (25%) | | 352 (20%) | |
| Europe | 539 (26%) | 118 (39%) | | 319 (18%) | |
| North America | 1154 (56%) | 107 (36%) | | 1103 (62%) | |
| Country |  |  |  |  |  |
| Australia | 380 (18%) | 76 (25%) | | 352 (20%) | |
| Canada | 541 (26%) | 14 (5%) | | 538 (30%) | |
| Denmark | 95 (5%) | - | | - | |
| Germany | 71 (3%) | 15 (5%) | | 70 (4%) | |
| Greece | 52 (3%) | 23 (8%) | | 29 (2%) | |
| Switzerland | 247 (12%) | 80 (27%) | | 220 (12%) | |
| United States of America | 613 (30%) | 93 (30%) | | 565 (32%) | |
| United Kingdom | 74 (4%) | - | | - | |

*Data given is median (interquartile range) or number (%).

Additionally, numbers (n= ) are given where information is missing for some patients. This missing information occurred because we did not mandate demographic characteristic (see Supplemental Text S1, Appendix 1), and because some centers provided age as quartiles rather than exact age.

Abbreviations: FN, fever in neutropenia.

# 4

**Table S3** Patient characteristics per study site.

|  | Bern (Switzerland) | Copenhagen (Denmark) | Frankfurt (Germany) | Melbourne (Australia) | Philadelphia (United  States) | SPOG  (Switzerland) | Stanford (United  States) | Thessaloniki (Greece) | Toronto (Canada) | UK (United Kingdom) | Vanderbilt (United  States) |
| --- | --- | --- | --- | --- | --- | --- | --- | --- | --- | --- | --- |
| Patients | 90 | 95 | 71 | 380 | 194 | 157 | 95 | 52 | 541 | 74 | 324 |
| Age at FN diagnosis, | 5 (3 to 12) | NA | NA | 6 (3.5 to | 11.5 (7.5 to |  | 7 (4 to 12) | NA | 6.5 (3.5 to | NA | 7 (3 to 13) |
| years |  |  |  | 11.5) | 15) |  |  |  | 12) |  |  |
| Sex |  |  |  |  |  |  |  |  |  |  |  |
| Female | 43 (48%) | 37 (39%) | NA | 174 (46%) | 88 (45%) | 69 (44%) | 38 (40%) | 32 (62%) | 255 (47%) | NA | 152 (47%) |
| Male | 47 (52%) | 58 (61%) |  | 206 (54%) | 106 (55%) | 88 (56%) | 57 (60%) | 20 (38%) | 286 (53%) |  | 172 (53%) |
| Type of malignancy |  |  |  |  |  |  |  |  |  |  |  |
| ALL | 46 (51%) | 46 (48%) | NA | 161 (42%) | 63 (32%) | 69 (44%) | 42 (44%) | 37 (71%) | 227 (42%) | 35 (47%) | 124 (38%) |
| AML | 4 (4%) | 18 (19%) | NA | 16 (4%) | 23 (12%) | 6 (4%) | 4 (4%) | 13 (25%) | 44 (8%) | 10 (14%) | 18 (6%) |
| HL | 2 (2%) | 2 (2%) | NA | 12 (3%) | 10 (5%) | 6 (4%) | 0 (0%) | 2 (4%) | 27 (5%) | 0 (0%) | 7 (2%) |
| NHL | 6 (7%) | 9 (9%) | NA | 17 (4%) | 5 (3%) | 18 (11%) | 4 (4%) | 0 (0%) | 32 (6%) | 0 (0%) | 29 (9%) |
| CNS tumor | 9 (10%) | 4 (4%) | NA | 35 (9%) | 34 (18%) | 16 (10%) | 6 (6%) | 0 (0%) | 47 (9%) | 13 (18%) | 23 (7%) |
| Other solid tumors | 23 (26%) | 14 (15%) | NA | 116 (31%) | 43 (22%) | 42 (27%) | 35 (37%) | 0 (0%) | 150 (28%) | 11 (15%) | 105 (32%) |
| Other malignancies | 0 (0%) | 2 (2%) | NA | 23 (6%) | 16 (8%) | 0 (0%) | 4 (4%) | 0 (0%) | 14 (3%) | 5 (7%) | 18 (6%) |
| Relapsed malignancy |  |  |  |  |  |  |  |  |  |  |  |
| Yes | 2 (2%) | NA | NA | 29 | 46 | 9 | 9 | 6 | 41 | NA | NA |
| No | 88 (98%) | NA | NA | 102 | 147 | 148 | 82 | 46 | 500 | NA | NA |
| FN episodes per | 2, max 7 | 2, max 8 | 2, max 9 | 2, max 9 | 1, max 11 | 2, max 6 | 1, max 4 | 1, max 2 | 1, max 10 | NA | 2, max 8 |
| patient (median) |  |  |  |  |  |  |  |  |  |  |  |
| FN episodes included | 204 | 222 | 211 | 780 | 354 | 347 | 137 | 53 | 922 | 74 | 702 |
| in study |  |  |  |  |  |  |  |  |  |  |  |
| Episodes with severe | 23 (26%) | NA | 16 (8%) | 90 (12%) | 46 (13%) | 27 (17%) | 43 (31%) | 23 (43%) | 14 (2%) | NA | 19 (3%) |
| disease at presentation |  |  |  |  |  |  |  |  |  |  |  |
| Episodes without | 67 (74%) | NA | 195 (92%) | 690 (88%) | 308 (87%) | 130 (83%) | 94 (69%) | 30 (57%) | 908 (98%) | NA | 683 (97%) |
| severe disease at |  |  |  |  |  |  |  |  |  |  |  |
| presentation |  |  |  |  |  |  |  |  |  |  |  |

Abbreviations: ALL, acute lymphoblastic leukemia; AML, acute myeloid leukemia; CNS, central nervous system tumor; FN, fever in neutropenia; HL, Hodgkin lymphoma; NHL; Non-Hodgkin lymphoma; SPOG, Swiss Pediatric Oncology Group.

# 5

**Table S4** Patient characteristics stratified by TTA ≤ 60 minutes versus > 60 minutes.*

|  | TTA ≤ 60 min |  | TTA > 60 min |
| --- | --- | --- | --- |
| Number of patients | 1996 |  | 2010 |
| Age at FN diagnosis, years | n = 1577  7.3 (3.4 to 12) |  | n = 1517  6.6 (3.6 to 12) |
| Sex  Female | n = 1788  810 (45%) |  | n = 1933  892 (46%) |
| Male | 978 (55%) |  | 1041 (54%) |
| Type of malignancy | n= 1836 |  | n = 1959 |
| Acute lymphoblastic leukemia | 741 (40%) |  | 858 (44%) |
| Acute myeloid leukemia | 154 (8%) |  | 145 (7%) |
| Hodgkin lymphoma | 37 (2%) |  | 59 (3%) |
| Non-Hodgkin lymphoma | 116 (6%) |  | 120 (6%) |
| Central nervous system tumor | 152 (8%) |  | 164 (8%) |
| Other solid tumors | 545 (30%) |  | 553 (28%) |
| Other malignancies | 91 (5%) |  | 60 (3%) |
| Relapsed malignancy | n = 921 |  | n = 1363 |
| Yes | 168 (18%) |  | 158 (12%) |
| No | 753 (82%) |  | 1205 (88%) |
| Continent |  |  |  |
| Australia | 500 (25%) |  | 280 (14%) |
| Europe | 532 (27%) |  | 579 (29%) |
| North America | 964 (48%) |  | 1151 (57%) |
| Location at fever detection | n = 1669 |  | n = 1673 |
| Outside the hospital | 1177 (71%) |  | 1195 (71%) |
| In the hospital | 492 (29%) |  | 478 (29%) |
| Bone marrow involvement** | n = 771 |  | n = 1228 |
| Yes | 118 (15%) |  | 208 (17%) |
| No | 653 (85%) |  | 1020 (83%) |
| CVAD  Yes | n = 1456  1388 (95%) |  | n = 1632  1368 (89%) |
| No | 68 (5%) |  | 264 (11%) |
| Max fever at diagnosis (°C)*** | n = 1479 |  | n = 1694 |
| median (IQR) | 38.7 (38.4 to 39.1) |  | 38.8 (38.4 to 39.3) |
| Leucocyte count (G/l) median (IQR) | n = 1521  0.4 (0.2 to 0.9) |  | n = 1789  0.6 (0.2 to 1.2) |
| Neutrophil count (G/l) | n = 1195 |  | n = 1248 |
| median (IQR) Hemoglobin (g/l) | 0.02 (0.00 to 0.14)  n = 1456 |  | 0.00 (0.06 to 0.24)  n = 1629 |
| median (IQR) | 84 (71 to 98) |  | 83 (70 to 97) |
| Platelet count (G/l) median (IQR) | n = 1461  40 (18 to 86) |  | n = 1688  51 (25 to 115) |
| Chemotherapy intensity | n = 321 |  | n = 398 |
| 1 | 70 (22%) |  | 91 (23%) |
| 2 | 117 (55%) |  | 261 (66%) |
| 3 | 43 (13%) |  | 34 (9%) |
| 4 | 31 (10%) |  | 12 (3%) |

*Data given is median (interquartile range) or number (%). Additionally, numbers (n = ) are given for information not reported in all patients. **Radiological or morphological bone marrow involvement.

***Indicating the highest temperature measured before diagnosis of FN.

Abbreviations: CVAD, central venous access device; FN, fever in neutropenia; ICU, intensive care unit; IQR, interquartile range. SMC, serious medical complication; SRE, safety relevant event; TTA, time from fever to start of antibiotics;

| **Table S5** FN episode characteristics.* |  | | | | |
| --- | --- | --- | --- | --- | --- |
|  | Total episodes in study |  | Episodes with severe disease at presentation |  | Episodes without severe disease at presentation |
| FN episodes | 4006 |  | 345 |  | 3365 |
| TTA, median (IQR) | 61 min (35 – 120) |  | 53 min (31 – 94) |  | 60 min (35 to 118) |
| TTA ≤ 60 min | 1996 (50%) |  | 191 (55%) |  | 1694 (50%) |
| TTA > 60 min | 2010 (50%) |  | 154 (45%) |  | 1671 (50%) |
| Outcomes Death | 35 (1%) |  | 9 (3%) |  | 20 (1%) |
| Bacteremia | 601 (15%) |  | 95 (28%) |  | 349 (10%) |
| ICU | 166 (4%) |  | 50 (15%) |  | 83 (2%) |
| SMC | 182 (5%) |  | 55 (16%) |  | 94 (3%) |
| SRE | 692 (17%) |  | 119 (35%) |  | 410 (12%) |
| Location at fever detection | n = 3342 |  | n = 280 |  | n = 3062 |
| Outside the hospital | 2372 (71%) |  | 206 (74%) |  | 2166 (71%) |
| In the hospital | 970 (29%) |  | 74 (26%) |  | 896 (29%) |
| Bone marrow involvement** | n = 1999 |  | n = 188 |  | n = 1811 |
| Yes | 326 (16%) |  | 32 (17%) |  | 294 (16%) |
| No | 1673 (84%) |  | 156 (83%) |  | 1517 (84%) |
| CVAD | n = 3088 |  | n = 319 |  | n = 2769 |
| Yes | 2756 (89%) |  | 308 (97%) |  | 2448 (88%) |
| No | 332 (11%) |  | 11 (3%) |  | 321 (12%) |
| Max fever at diagnosis (°C)*** | n = 3173 |  | n = 276 |  | n = 2685 |
| median (IQR) | 38.7 (38.4 to 39.2) |  | 38.8 (38.5 to 39.3) |  | 38.7 (38.4 to 39.2) |
| Leucocyte count (G/l) | n = 3310 |  | n = 319 |  | n = 2769 |
| median (IQR) | 0.5 (0.2 to 1.0) |  | 0.4 (0.14 to 0.9) |  | 0.5 (0.2 to 1.1) |
| Neutrophil count (G/l) | n = 3173 |  | n = 319 |  | n = 2033 |
| median (IQR) | 0.03 (0.00 to 0.20) |  | 0.04 (0.00 to 0.2) |  | 0.04 (0.00 to 0.21) |
| Hemoglobin (g/l) | n = 3085 |  | n = 318 |  | n = 2767 |
| median (IQR) | 83 (70 to 98) |  | 84 (71 to 98) |  | 83 (70 to 97) |
| Platelet count (G/l) | n = 3149 |  | n = 286 |  | n = 2643 |
| median (IQR) | 46 (21 to 100) |  | 43 (20 to 87) |  | 48 (22 to 104) |
| Chemotherapy intensity | n = 719 |  | n = 125 |  | n = 594 |
| 1 | 161 (22%) |  | 13 (10%) |  | 148 (25%) |
| 2 | 438 (61%) |  | 72 (58%) |  | 366 (62%) |
| 3 | 77 (11%) |  | 21 (17%) |  | 56 (9%) |
| 4 | 43 (6%) |  | 19 (15%) |  | 24 (4%) |
| Season | n = 3525 |  | n = 276 |  | n = 3027 |
| Winter (Dec – Mar) | 1215 (34%) |  | 103 (37%) |  | 1035 (34%) |
| No winter (Apr – Nov) | 2310 (66%) |  | 173 (63%) |  | 1992 (66%) |
| Time of presentation | n = 3513 |  | n = 276 |  | n = 3015 |
| Nighttime (20:00 – 07:59) | 1494 (43%) |  | 108 (39%) |  | 1303 (43%) |
| Daytime | 2019 (57%) |  | 168 (61%) |  | 1712 (57%) |
| Weekend (Sat/Sun) | 913 (26%) |  | 69 (25%) |  | 792 (26%) |
| Other day | 2600 (74%) |  | 207 (75%) |  | 2223 (74%) |
| Out of office time (weekend or | 2001 (57%) |  | 148 (54%) |  | 1737 (58%) |
| night) |  |  |  |  |  |
| Office time | 1512 (43%) |  | 128 (46%) |  | 1278 (42%) |

*Data given is median (interquartile range) or number (%). Additionally, numbers (n = ) are given for information not reported in all patients.

. **Radiological or morphological bone marrow involvement. ***Indicating the highest temperature measured before diagnosis of FN. Abbreviations: CVAD, central venous access device; FN, fever in neutropenia; ICU, intensive care unit; IQR, interquartile range. SMC, serious medical complication; SRE, safety relevant event; TTA, time from fever to start of antibiotics;

**Table S6** Exploratory analyses: Association between time from arrival at the hospital to start of antibiotics (TTA) and the occurrence of safety relevant events (SRE) corrected for 1) the inverted proportion of FN episodes with severe disease at presentation, 2) a propensity score and 3) including location at fever detection and the interaction of TTA with this location as additional regression parameters.

1) **Results of two-level mixed logistic**

**regression***

|  | **345 FN episodes** | **119 SRE** | **Odds ratio (95% CI)** | **P-value** |
| --- | --- | --- | --- | --- |
| **Binary variable**  ≤60min | 191 (55%) | 78 (66%) | 1 (Reference) | - |
| >60min | 154 (45%) | 41 (34%) | 0.53 (0.28 to 0.99) | 0.048 |

2) **Results of three-level mixed logistic**

**regression with propensity score****

|  | **188 FN episodes** | **63 SRE** | **Odds ratio (95% CI)** | **P-value** |
| --- | --- | --- | --- | --- |
| **Binary variable**  ≤60min | 101 (54%) | 36 (57%) | 1 (Reference) | - |
| >60min | 87 (46%) | 27 (43%) | 0.82 (0.41 to 1.66) | 0.58 |

3) **Results of three-level mixed logistic**

**regression including location at fever detection and an interaction term between location and TTA as regression parameters**

|  | **280 FN episodes** | **105 SRE** | **Odds ratio (95% CI)** | **P-value** |
| --- | --- | --- | --- | --- |
| **Binary variable**  ≤60min | 166 (59%) | 69 (66%) | 1 (Reference) | - |
| >60min | 114 (41%) | 36 (34%) | 0.96 (0.35 to 2.62) | 0.931 |

Abbreviations: CI, confidence interval; FN, fever in neutropenia; SRE, safety relevant event.

* The inverse proportion of episodes with severe disease at presentation are defined per dataset, which is equivalent in most datasets with the definition of country. So here two-level instead of three-level mixed logistic regression, with random intercepts per patient (not nested within per-country intercepts) was used.

** A propensity score balancing the probability of treatment assignment with observed baseline variables developed in an earlier study on TTA [Koenig et al. Sci Rep. 20222; Ref. 14 of the manuscript] was calculated as continuous variable for each episode.

**Research plan/Protocol for HRO:**

**Further use of biological material and health-related personal data for research pursuant to Articles 32 and 33 HRA**

**Title of the research project**

Association of time to antibiotics (TTA) with outcome in children undergoing chemotherapy for cancer with fever in neutropenia (FN) – Umbrella Network Project 2022.1: International TTA Individual patient data (IPD) Meta-analysis

## Name and address of the project leader

Dr. med. Christa Koenig, MD, PhD, Physician, Pediatric Hematology/Oncology, Department of Pediatrics, Inselspital, Bern University Hospital, 3010 Bern, Switzerland, +41 31 632 21 11, [christa.koenig@insel.ch](mailto:christa.koenig@insel.ch)

## Confirmation of the project leader and sponsor

With my signature, I attest that all information in this protocol is correct and that I will comply with the information I have given and with national legislation, namely data protection law.


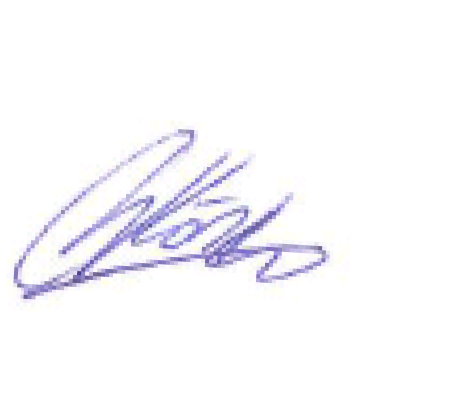
Project leader/Sponsor: Christa Koenig

Biel, 22.12.2023

| Place, date | Signature |
| --- | --- |

## Name and address of the project vice leader


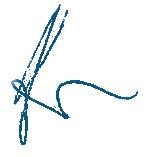
Prof Dr. med. Roland A Ammann, MD, Physician and DAS in Applied Statistics, Senior consultant Inselspital, StatConsult Ammann, Pleerweg 92, 3400 Burgdorf, +41 79 577 14 11, [info@statconsultammann.ch](mailto:info@statconsultammann.ch), [roland.ammann@hin.ch](mailto:roland.ammann@hin.ch)

Project vice leader: Roland A. Ammann

Burgdorf, 22.12.2023 Place, date Signature

| **Study Committee** |  | |
| --- | --- | --- |
| Christa Koenig, MD, PhD | [christa.koenig@insel.ch](mailto:christa.koenig@insel.ch) | Physician |
| Eva Brack, MD, PhD | [eva.brack@insel.ch](mailto:eva.brack@insel.ch) | Ped. Oncologist |
| Roland A Ammann, MD | [info@statconsultammann.ch](mailto:info@statconsultammann.ch) | Ped. Oncologist |

This study will be conducted by members of the Umbrella Network, an international collaboration which focuses on clinical research on infectious complications in pediatric cancer patients.

## Abbreviations

CRF case report form

CSV comma-separated values

FN fever in neutropenia

HRA Human Research Act

HRO Human Research Ordinance

IPD individual patient data

PI principal investigator

SMC serious medical complication

SRE safety relevant events

TTA time to antibiotics

## Background

- 1. **Fever in neutropenia (FN)**

Children and adolescents with cancer receive intensive chemotherapy that induces neutropenia. Neutropenia is one of the major risk factors for infections during cancer treatment. During neutropenia the natural immune response to inflammation is limited and signs and symptoms of infection may be obscured [1] Fever is often the only sign of an infection. Due to the risk of a complicated clinical course, fever in neutropenia (FN) is treated as a medical emergency.

Emergency administration of empirical broad-spectrum intravenous antibiotics is recommended by current FN guidelines [2-4]. But the nature of FN is heterogeneous and a pathogen, including bacteria found in the blood (bacteremia), can be identified in only about a quarter of FN episodes [5, 6].

## 1.2 Time to antibiotics (TTA) in FN

Time to antibiotics (TTA) refers to the time span between first recognition of fever or arrival at the hospital, and start of empiric intravenous antibiotic administration [7]. Although it seems intuitive that longer TTA affects clinical and safety outcomes, results are inconsistent [8].

Despite insufficient evidence, a TTA ≤60 min is often used as quality of care measure [9]. Correspondingly, several studies have used considerable resources to reduce TTA [10], presuming that there is a beneficial effect on clinical and safety outcomes. But before we can make evidence-based recommendations for TTA target times and use considerable resources for its reduction, we have to know whether a shorter TTA, i.e., earlier antibiotic treatment really reduces infections and their complications, and thus clinical and safety outcomes.

## Current evidence regarding effect of TTA on outcomes in FN

Today, there are hints, but not conclusive evidence, that at least two groups of FN patients should be distinguished: First, patients with severe disease at FN presentation benefit from short TTAs, which calls for aiming at TTAs of ≤60minutes, and maybe even shorter TTAs, despite the resources needed to reach these aims. Second, patients who are clinically well at FN presentation, do not benefit from these short TTAs.

The distinction of these groups is non-trivial, mainly because of the important effect of triage bias. Triage bias refers to the fact that patients who clinically have severe disease at FN presentation are treated faster by the emergency teams. This leads to the counterintuitive finding that a shorter TTA is associated with worse outcomes in analyses not corrected for this triage bias [8, 11]. Analyses can be corrected for triage bias by using covariates or covariate scores, or stratifying analyses to groups of FN episodes with severe versus non-severe diasease at FN presentation. In any case, such analyses need large numbers of FN episodes studied [8, 11]

## Clinical implications of potentially improved evidence

Should the hints mentioned above be replaced by evidence, this would have relevant clinical implications. First, resources used in the emergency department for FN patients could be reduced, because patients who are clinically well at FN presentation could be treated with lower priority, without the short TTA aim. Second, this would allow as well to spare antibiotics, because waiting for neutrophil count results would be possible in these patients before start of empiric broad-spectrum antibiotic treatment. This would lead to avoiding such treatment in patients with fever without neutropenia, considering that this therapy is associated with resistance, costs and toxicity [12, 13].

## Conclusion, outlook and study aim

In conclusion, there is insufficient evidence based on prospectively collected data regarding the effect of TTA on outcomes in children with FN. There is still less evidence from analyses correcting for triage bias.

From an ethical point of view, randomized controlled trials are not possible for the direct randomized controlled investigation of high versus low TTA on FN outcome. But large observational studies based on high-quality data may generate high-quality evidence as well. This study aims to use individual patient data (IPD), collected prospectively by different pediatric oncology centers in different middle- to high-income countries in different continents, to perform an IPD meta-analysis of the association of TTA on outcomes in pediatric patients with FN.

1. **Hypothesis, objectives, endpoints and definitions**

## Primary Hypothesis

We hypothesise that a longer TTA is associated with more safety relevant events (SRE) in children and adolescents undergoing chemotherapy for cancer who have severe disease at presentation with FN.

## Primary objective and endpoint

The primary objective of this analysis is to investigate the association between TTA and SRE in children and adolescents undergoing chemotherapy for cancer who have severe disease at presentation with FN.

The corresponding primary endpoint is the presence versus absence of an SRE in the respective FN episode, as defined below in 2.4.

## Secondary objectives and endpoints

Secondary objectives, comparable to the primary objective, are to investigate the association between TTA and SRE

1. in all pediatric patients undergoing chemotherapy for cancer presenting with FN (with or without severe disease at FN presentation)
2. according to the patients’ location at fever detection (presenting in the hospital versus outside the hospital)

Additional secondary objectives are:

1. to investigate the associations mentioned above in 2.1 and 2.2.A/B based on TTA as time from fever detection to start of antibiotics

The corresponding secondary endpoint for A), B) and C) is the presence versus absence of an SRE in the respective FN episode, as defined below in 2.4.

1. to assess the associations mentioned above in 2.1 and 2.2.A/C with the SRE composites (bacteremia, SMC, death or admission to an intensive care unit) as separately analyzed outcomes.

The corresponding secondary endpoints for D) is the presence versus absence of bacteremia, SMC, death and admission to an intensive care unit in the respective FN episode, as defined below in 2.4.

## Definitions

**Time to antibiotics (TTA)** is defined as the time from arrival at the hospital to start of antibiotics. Only for the secondary objective C, TTA will be defined as time from fever detection to start of antibiotics.

**A safety relevant event (SRE)** is defined as bacteremia detected during FN, and/or a serious medical complication (SMC) occurring during FN.

**Bacteremia** is defined as the culture of a recognized pathogen from at least one blood culture. This includes viridans group streptococci in the setting of concomitant mucosal barrier injury [14].

A **Serious Medical Complication (SMC)** is defined according to a modified consensus definition [15] as death due to any cause during FN, or admission to intensive care unit, high dependency unit or other critical care unit for organ support.

The modification here consists in leaving out severe sepsis (including septic shock) as an SMC defining event. This is due to multiple reasons:

- - - First, there is a relevant discrepancy between the current research definition of septic shock [16]– soon to be replaced – and the clinical use of this term. Patients clinically diagnosed with severe sepsis, according to non -standardized in-house definitions, will usually require treatment in an intensive care unit, high dependency unit or other critical care unit for organ support. Their FN episode will thus be classified as SMC here anyway.
    - Second, this discrepancy is further increased in the setting of chemotherapy and FN, because both fever and neutropenia are components of the definition of septic shock. The same is true for other direct effects of chemotherapies frequently used in pediatric oncology, like increased liver enzymes, and disturbed coagulation parameters [16]. This can lead to the counterintuitive situation that a patient with FN who is clinically well, but has abnormal laboratory parameters known to be directly due to chemotherapy and known to return to normal without any intervention, fulfills the research criteria of severe sepsis.
    - Third, the definitions used clinically for severe sepsis vary from institution to institution, reflecting the missing clinical usefulness of the research criteria. Thus, a consistent definition is not possible in the setting of this retrospective multicenter study.
    - Fourth, information on clinically defined severe sepsis is not routinely collected prospectively (non-published information collected while preparing this study).

**Severe disease at FN presentation** is defined as severe sepsis or reduced clinical condition (i.e., a general deterioration in physical health) at presentation with FN, assessed at first contact by the responsible physician.

Definitions described here are used for FN episodes collected within Switzerland. For data contributed by centers from outside Switzerland, definitions may slightly differ, but will only be used for analyses when considered as comparable and clinically sensible by the study team.

## Design

This is an international multicenter study IPD meta-analysis, i.e., a retrospective analysis on prospectively collected data. Investigated data includes information on patient and disease characteristics, laboratory blood values and clinical presentation, TTA, initial antimicrobial treatment and FN outcome. All data arise from pediatric patients with cancer undergoing chemotherapy treated within one of the contributing centers.

The international study center is located at the Department of Pediatric Hematology/Oncology, Inselspital, Bern University Hospital, in Bern, Switzerland.

The main data from Switzerland was collected during the prospective SPOG 2015 FN Definition study (NCT02324231) between April 28, 2016 and August 27, 2018 in 6 Swiss pediatric oncology centers (Basel, Bern, Geneva, Lausanne, Lucerne, Zurich) [17].

Data from September 2018 to December 2023 will be collected from Bern only.

Data from centers outside Switzerland will be collected for patients presenting with FN from January 01, 2016 to December 31, 2023.

Thus, these data will be retrospectively collected and analyzed, using, however, only data collected prospectively and stored electronically for routine clinical use, for use in data quality measures like certifications, or within prospective clinical studies.

No personal or identifying data will be collected for this study. Either anonymized or encoded data will be used, according to the respective local legal requirements. In case of encoded data, the key to re-link the data to a patient will not be shared with the study center in Bern, but will be kept in the respective study center.

## Origin of the data/biological material

All data stem from pediatric patients with cancer undergoing chemotherapy treated within one of the contributing centers, and presenting with FN.

Data from the SPOG 2015 FN Definition study [17] is retrieved from the study database. This data was prospectively collected on paper case report forms (CRFs) either by the principal investigator (PI) or a designated representative authorized by the PI. Authorization of any local staff member to make CRF entries was documented on the according staff list. The information from the paper CRFs was then transferred to a REDCap database in the study center in Bern. The source documents, including the patients’ charts and the paper CRFs, will not be used for this analysis. For this study only one additional variable (triage category assigned prospectively by the emergency team) will be collected additionally per center and retrieved retrospectively from electronic data records of the participating centers.

Of course, eligibility and exclusion criteria of this study, as detailed in chapters 5 and 6, for patients and FN episodes of the SPOG 2015 FN Definition study apply as well. The respective criteria of this study do not exactly match the respective criteria of the SPOG 2015 FN definition study. Correspondingly, a minority of patients and of FN episodes will not be eligible for this study. And the SMC and thus the SRE outcome differ between the studies because severe sepsis is not a SMC defining criterion.

Further data from Bern and centers abroad were collected prospectively and stored electronically for routine clinical use, for use in treatment quality measures including certifications, or within prospective clinical studies. These data will be extracted from electronic patients’ charts, from the quality measure databases, or from the clinical study databases, respectively, by local researchers.

In Bern, a designated member of the research team will screen all pediatric oncology patients treated between September 2018 to December 2023, for eligibility. For patients fulfilling the inclusion criteria, FN episodes will be identified by screening the patients list of diagnoses and discharge letters.

A summary of variables minimally required and of additional variables desired is available in Appendix 1. This summary, together with this study protocol and the response of the Cantonal Ethics Committee of Bern is shared with all contributing centers.

In the study center in Bern, all data from contributing centers will be checked for quality and plausibility. Data passing these checks will then be merged into a .rda datafile using the R software [18]. The final .rda file will be used to generate an additional .csv file with comma- separated values, which is usable by many software programs. Both files will be stored according to current legislation.

No information on names, initials and dates of birth will be stored in these data files. A combination of site acronym and participant number will be used to be able to identify multiple FN episodes per patient and center.

## Inclusion criteria

- 1. **Centers**

Contributing centers outside Switzerland have to ensure that shared data was collected according to the respective national and local regulations. Retrospective collection of data may be exempted from the need for formal ethic committee approval, but researchers are advised to have protocol reviews by ethic boards.

Centers have to provide a minimum set of mandatory variables per center to participate in this study (see also Appendix 1), otherwise these centers are not eligible for any participation in this study:

## Mandatory variables per center

| **Mandatory variables** | |
| --- | --- |
| **Variable** | **Attributes** |
| Study center | Text |
| Definition of neutropenia used for FN | Real number (_ _ . _ G/L) |
| Definition of fever limit used for FN | Real number (_ _ . _°C) |
| Definition for death | Text |
| Definition for bacteremia | Text |

These definition may slightly differ between contributing centers. Data will only be used for this study when definitions are considered as essentially comparable to definitions described above (2.3) and as clinically sensible judged by the project leader and the vice project leader. The respective definitions, and thus these differences, will be described in the study reports and the respective publications.

## Patients

Pediatric patients younger than 18 years (at presentation with FN) undergoing chemotherapy for cancer for any malignancy and presenting with FN.

Centers have to provide a minimum set of mandatory variables per patient, otherwise these patients and all their respective FN episodes are not eligible for this study.

## Mandatory variables per patient

| **Mandatory variables** | |
| --- | --- |
| **Variable** | **Attributes** |
| Center specific patient identifier | Text |
| Treated with chemotherapy for cancer | any binary coding (number or text) |

- 1. **FN episodes**

Centers have to provide a minimum set of mandatory variables per FN episode, otherwise these FN episodes are not eligible for this study.

## Mandatory variables per FN episode

| **Times: either TTA (min/hour) or times (arrival and start of AB)** | |
| --- | --- |
| **Variable** | **Attributes** |
| Time to antibiotics (arrival to start) | Integer ( minutes) or other format |
| Time of arrival at the hospital/emergency room | Date and time (any format ) |
| Time of start of first dose of antibiotics | Date and time (any format ) |

| **If none of the above information on TTA is available this is the minimal information needed:** | |
| --- | --- |
| TTA (arrival to start) ≤ 60 min | Any binary coding (number or tet) |
| **Outcomes per FN episode** | |
| Death | any binary coding (number or text) |
| Bacteremia | any binary coding (number or text) |
| Admission to intensive care unit | any binary coding (number or text) |

We are aware that centers providing only the mandatory variables cannot be included for the primary analysis. We deliberately did not include the variable defining severe disease at presentation with FN as mandatory variable but as “variables additionally needed for primary analysis”, to be able to collect a larger dataset. Additionally, we believe that we will get more accurate information for severe disease at presentation by using this approach.

## Variables additionally needed for primary analysis

| **At least one of 3 variables defining “severe disease at presentation with FN”** | |
| --- | --- |
| **Variable** | **Attributes** |
| Severely reduced general condition at presentation | any binary coding (number or text) |
| Sepsis at presentation | any binary coding (number or text) |
| Triage category assigned by emergency team | Text or number |

Additional variables desired but not mandatory for study participation, are outlined in the Appendix 1.

## Exclusion criteria

- 1. **Centers**
     - Insufficient data on mandatory variables per center (see 5.1).

## Patients

- - - Refused (general) informed consent by the patients and/or their legal guardians, or comparable non-consent situation according to local legislation.
    - Insufficient data on mandatory variables per patient (see 5.2)
    - Not meeting inclusion criteria (see 5.2)

## FN episodes

- - - Insufficient data on mandatory variables per FN episode (see 5.3)

## Information and consent of participants

Patients from Switzerland, participating in the SPOG 2015 FN Definition study, signed an informed consent including further use of encrypted data for research purposes (Appendix 2).

Additional patients from Switzerland (Bern) and/or their legal guardians have signed the general consent of the university hospital of Bern, for further use of clinical data (Appendix 3).

The informed consent process for the SPOG 2015 FN Definition study and the general consent is carried out according to international guidelines (ICH-GCP) and with special considerations for consenting vulnerable patients (children).

Centers outside Switzerland have to ensure that shared patient data is collected according to the respective national and local, legal and ethical regulations. Retrospective collection of data may be exempted from the need for formal ethic committee approval, but researchers are advised to have protocol reviews by ethic boards.

## Scientific methods and sample size

- 1. **Power calculation**

A power analysis was made using the „power.prop.test“ function from the „pwr“ library in R [19], based on the proportions of 44% (4 of 9) FN with SRE at TTA ≤60 min versus 60% (16 of 27) with SRE at TTA > 60 min reported in [11]. This power analysis led to a sample size of 2x165 episodes of FN with severe disease at presentation to reach a power of 90% to detect an increase of proportions from 44% to 60% at a one-sided alpha of 0.05.

Unequal sample sizes, which are expected here, will somewhat decrease this power. This is why the power analysis aimed at a power of 90% instead of 80%.

Assuming that 13.5% of all FN episodes are with severe disease at presentation [11], the sample size of 330 FN episodes with severe disease at presentation correspond to about 2440 FN episodes in total. The study sites that have confirmed their participation until date of submission intend to report > 5500 FN episodes in total (Appendix 4). Assuming that the information on disease severity at presentation with FN is available in 75% of FN episodes, which corresponds to > 4125 FN episodes, the target sample size of totally 2440 FN episodes with this information is expected to be more than reached.

## Statistical analysis plan

Descriptive statistic using standard methods will be performed.

## Primary objective

For the primary objective, two analyses of the association between TTA (arrival at the hospital to start of antibiotics) and SRE will be performed in the subset of patients with severe disease at presentation with FN.

First, TTA will be analyzed as binary variable (≤ 60 min versus > 60 min), and logistic three-level mixed regression with random intercepts per patient, nested within country, to account for multiple episodes per patient, will be done.

Second, TTA will be analyzed as categorical variable with logistic three-level mixed regression with random intercepts per patient, nested within country. This analysis will be performed in those patients with severe disease at FN presentation, for whom the TTA is known quantitatively, not only as binary variable ≤ versus >60 minutes.

For this analysis, the adjacent categories method [20, 11] will be used to identify time intervals with significantly different risk of SRE. At start of this analysis, intervals of 15min will be used up to 120min and 30min intervals up to 360min. At the end of this analysis, two or more time intervals with significantly different risk of SRE are expected.

## Secondary objectives

Analyses comparable to the primary analysis will be done for the following patient groups:

1. All patients presenting with FN; and patients presenting without severe disease (TTA: binary and categories as identified above)
2. Stratified according to location at FN diagnosis: inpatients versus not in study site (e.g. at home) (Patients: all patients, patients with and patients without severe disease at presentation; TTA: binary and categories as identified above in 8.2.1)

Analyses comparable to the primary analysis will be done for:

1. Alternative TTA definition: Time from fever recognition to start of antibiotics (Patients: all patients, patients with and patients without severe disease at presentation; TTA: binary and categories identified by the adjacent categories method)
2. Components of primary outcome:
   1. Serious medical complications (binary outcome) (Patients: all patients, patients with and patients without severe disease at presentation TTA: binary and categories as identified above)
   2. Bacteremia (binary outcome) (Patients: all patients, patients with and patients without severe disease at presentation TTA: binary and categories as identified above)

The current version of the R software [18] will be used for analysis.

## Correction for biases

Triage bias, e.g. faster treatment of patients presenting in reduced general condition or at higher risk for poor outcome, is an important confounding factor when analyzing TTA [8, 11]. Without bias correction, the true effect of TTA on patient important outcomes may be undetectable.

Our analyses correct for this bias by stratifying the analysis for patients with versus without severe disease at FN presentation (stratified analysis).

## Handling of missing data, exclusion of data

There will be no imputation for missing data. Data will only be used for the study when the respective relevant definitions are comparable to the definitions described above (2.3) and clinically sensible judged by the PI. Differences in definitions will be described and discussed in the resulting manuscript.

## Reporting obligations

The ethics committee must be notified of any change of the project leader in advance. The completion or discontinuation of the research project must be reported to the ethics committee within 90 days.

## Data protection

**Uncoded data, coding and storage of the key**

Data handling and protection is conducted according to hospital data-privacy SOPs, ICH-GCP and applicable regulations. Data collected from the hospital patient charts are considered the source data and will be stored in the respective study center.

Data from the SPOG 2015 FN Definition study is retrieved only in an encoded form from the study databased (RedCap). For additional data, retrospectively collected in Switzerland (Bern), a member of the study team will extract the data from the patients’ charts into an Excel sheet. A key document with which the data can be assigned to patients will be made. The key will be stored in the study center. In other contributing centers, comparable methods will be used at the discretion of the local study PIs.

Despite this, only anonymized or encoded data will be handled for this study. If uncoded data is shared by contributing centers, these will be destroyed. If encoded data is shared, the key will be kept at a secure place in the contributing center and is not shared with the study team. When entered in the study database, a combination of site acronym and a neutral patient number will be used.

In the resulting publications, identification of participants will be impossible.

## Information on the storage of data and samples

For this project only anonymized or encoded data will be handled. Data collected will be stored electronically.

## Retention period

Storage of source data will be done at the respective centers. The participating study centers are responsible to meet the local regulatory and legal requirements for collected data storage. For Switzerland, source documents and documents pertaining to the conduct of this study, including CRFs, are archived for a minimum of 10 years after the completion or premature termination of the study.

All anonymised or encoded data collected for this study will be stored as .rda and .csv files for a minimum of 10 years.

## Ethical and regulatory requirements

This protocol seeks approval for the use of Swiss patient data and the analyses planned. For data from abroad, the contributing centers and their respective local PIs will ensure that the respective national and local, legal and ethical regulations are met before transmission of data.

This is a retrospective study on prospectively collected data. Therefore this study does not place a risk for the patients and their treatment. The main risk associated with the participation in the study is loss of privacy. To minimize this risk, strict adherence to data safety and hospital data confidentiality SOPs is guaranteed by the PI. Further, the study will be implemented according to current valid international guidelines (ICH-GCP). The ethical position is based on the Declaration of Helsinki [21], thus guaranteeing optimal protection of patient interests.

Results of this project may benefit pediatric patients undergoing chemotherapy for cancer presenting with FN, as it will help to improve management of FN.

Other than being included in manuscript writing and co-authorship, there is no specific benefit for local PIs.

This project complies with the regulatory requirements of the HRA and the HRO. The prerequisite for carrying out the research project is the approval of the competent ethics committee.

## Results / transparency / publication

The use of IPD and the combination of international data will ensure generalization of the results for most pediatric patients undergoing chemotherapy for cancer. The detailed study results will be submitted for publication in peer-reviewed journals. Only anonymized data will be published.

## Funding / Data sharing / Declaration of interest

Funding for this study will be searched by grant applications to foundations and institutions granting support for clinical research. Such granting bodies will have no influence on study design, data collection, analysis, interpretation, decision to publish, or preparation of the manuscript.

The detailed study results will be submitted for publication in peer-reviewed journals. The current version of the ICMMJE recommendations regarding authorship eligibility is applicable.

The use of professional writers is not planned. Researchers collecting and providing data from centers outside Switzerland will be included in the manuscript writing process and offered co- authorship if applicable. The protocol is not confidential; it is intended to be made publicly available. There is no conflict of interest for the Sponsor-Investigator (project leader) and for the vice leader.

## References

1. Bodey GP, Buckley M, Sathe YS, Freireich EJ: Quantitative relationships between circulating leukocytes and infection in patients with acute leukemia. Ann Intern Med 1966, 64(2):328-340.
2. Lehrnbecher T, Averbuch D, Castagnola E, Cesaro S, Ammann RA, Garcia-Vidal C, Kanerva J, Lanternier F, Mesini A, Mikulska M et al: 8th European Conference on Infections in Leukaemia: 2020 guidelines for the use of antibiotics in paediatric patients with cancer or post-haematopoietic cell transplantation. Lancet Oncol 2021, 22(6):e270- e280.
3. AWMF S2K Guidelines: Diagnostik und Therapie bei Kindern mit onkologischer Grunderkrankung, Fieber und Granulozytopenie (mit febriler Neutropenie) außerhalb der allogenen Stammzelltransplantation [<http://www.awmf.org/uploads/tx_szleitlinien/048-> 014l_S2k_onkologische_Grunderkrankung_Fieber_Granulozytopenie_2016-04.pdf]
4. Lehrnbecher T, Robinson PD, Ammann RA, Fisher B, Patel P, Phillips R, Beauchemin MP, Carlesse F, Castagnola E, Davis BL et al: Guideline for the Management of Fever and Neutropenia in Pediatric Patients With Cancer and Hematopoietic Cell Transplantation Recipients: 2023 Update. J Clin Oncol 2023:Jco2202224.
5. Castagnola E, Fontana V, Caviglia I, Caruso S, Faraci M, Fioredda F, Garre ML, Moroni C, Conte M, Losurdo G et al: A prospective study on the epidemiology of febrile episodes during chemotherapy-induced neutropenia in children with cancer or after hemopoietic stem cell transplantation. Clin Infect Dis 2007, 45(10):1296-1304.
6. Ammann RA, Bodmer N, Hirt A, Niggli FK, Nadal D, Simon A, Ozsahin H, Kontny U, Kuhne T, Popovic MB et al: Predicting adverse events in children with fever and chemotherapy-induced neutropenia: the prospective multicenter SPOG 2003 FN study. J Clin Oncol 2010, 28(12):2008-2014.
7. Koenig C, Morgan J, Ammann RA, Sung L, Phillips B: Protocol for a systematic review of time to antibiotics (TTA) in patients with fever and neutropenia during chemotherapy for cancer (FN) and interventions aiming to reduce TTA. Systematic Reviews 2019, 8(1):82.
8. Koenig C, Schneider C, Morgan JE, Ammann RA, Sung L, Phillips B: Association of time to antibiotics and clinical outcomes in patients with fever and neutropenia during chemotherapy for cancer: a systematic review. Support Care Cancer 2019.
9. McCavit TL, Winick N: Time-to-antibiotic administration as a quality of care measure in children with febrile neutropenia: a survey of pediatric oncology centers. Pediatr Blood Cancer 2012, 58(2):303-305.
10. Koenig C, Schneider C, Morgan JE, Ammann RA, Sung L, Phillips B: Interventions aiming to reduce time to antibiotics (TTA) in patients with fever and neutropenia during chemotherapy for cancer (FN), a systematic review. Support Care Cancer 2019.
11. Koenig C, Kuehni CE, Bodmer N, Agyeman PKA, Ansari M, Roessler J, von der Weid NX, Ammann RA: Time to antibiotics is unrelated to outcome in pediatric patients with fever in neutropenia presenting without severe disease during chemotherapy for cancer. Sci Rep 2022, 12(1):14028.
12. Teuffel O, Amir E, Alibhai SM, Beyene J, Sung L: Cost-effectiveness of outpatient management for febrile neutropenia in children with cancer. Pediatrics 2011, 127(2):e279-286.
13. Morgan JE, Cleminson J, Atkin K, Stewart LA, Phillips RS: Systematic review of reduced therapy regimens for children with low risk febrile neutropenia. Support Care Cancer 2016, 24(6):2651-2660.
14. Centre for Disease Control and Prevention (CDC). Central Line-Associated Bloodstream Infection (CLABSI) Event (https://[www.cdc.gov/nhsn/pdfs/pscmanual/4psc_clabscurrent.pdf)](http://www.cdc.gov/nhsn/pdfs/pscmanual/4psc_clabscurrent.pdf))
15. Haeusler GM, Phillips RS, Lehrnbecher T, Sung L, Ammann RA: The reporting of outcomes in studies of fever and neutropenia in children with cancer: time for consensus. Pediatr Blood Cancer 2013, 60(10):1563-1564.
16. Goldstein B, Giroir B, Randolph A, International Consensus Conference on Pediatric S. International pediatric sepsis consensus conference: definitions for sepsis and organ dysfunction in pediatrics. Pediatric critical care medicine : a journal of the Society of Critical Care Medicine and the World Federation of Pediatric Intensive and Critical Care Societies 2005;6:2-8.
17. Koenig C, Bodmer N, Agyeman PKA, Niggli F, Adam C, Ansari M, Eisenreich B, Keller N, Leibundgut K, Nadal D et al: 39.0 degrees C versus 38.5 degrees C ear temperature as fever limit in children with neutropenia undergoing chemotherapy for cancer: a multicentre, cluster-randomised, multiple-crossover, non-inferiority trial. Lancet Child Adolesc Health. 2020, 4(7):495-502.
18. R Core Team: R: A language and environment for statistical computing. Vienna, 2014, (<http://www.R-project.org/)>
19. Champely S, Ekstrom C. Dalgaard P, Gill J, Weibelzahl S: Package “pwr”, Power analysis functions along the lines of Cohen (1988), 2022, ([https://CRAN.R-](https://cran.r/) project.org/package=pwr)
20. Altman, D. Practical Statistics for Medical Research (Chapman & Hall, 1991)
21. Declaration of Helsinki, Version October 2013, (<https://www.wma.net/policies-post/wma-> declaration-of-helsinki-ethical-principles-for-medical-research-involving-human-subjects/)

**Appendix 1**

**List of mandatory and additional desired variables:**

1. **Definitions**

| **1.1 Mandatory variables** | |
| --- | --- |
| **Variable** | **Attributes** |
| Study center | Text |
| Definition of neutropenia used for FN | Real number (_ _ . _ G/L) |
| Definition of fever limit used for FN | Real number (_ _ . _°C) |
| Definition for death | Text |
| Definition for bacteremia | Text |

| **1.2 Definitions of additional (desired) per patient variables, if applicable** | |
| --- | --- |
| **Variable** | **Attributes** |
| Definition of severely reduced general condition at presentation | Text |
| Definition of sepsis at presentation | text |
| Triage categories used | Text |
| Definition of chemotherapy intensities | Text |
| Definition for severe sepsis | Text |

1. **Mandatory variables per patient**

| **2.1 Mandatory variables per patient** | |
| --- | --- |
| **Variable** | **Attributes** |
| Center specific patient identifier | Text |
| Treated with chemotherapy for cancer | any binary coding (number or text) |

1. **Mandatory variables per FN episode**

| **3.1 Times: either TTA (min/hour) or times (arrival and start of AB)** | |
| --- | --- |
| Time to antibiotics (arrival to start) | Integer ( minutes) or other format |
| Time of arrival at the hospital/emergency room | Date and time (any format ) |
| Time of start of first dose of antibiotics | Date and time (any format ) |
| **If none of the above information on TTA is available this is the minimal information**  **needed:** | |
| TTA (arrival to start) ≤ 60 min | Any binary coding (number or tet) |
| **3.2 Outcomes per FN episode** | |
| Death | any binary coding (number or text) |
| Bacteremia | any binary coding (number or text) |
| Admission to intensive care unit | any binary coding (number or text) |

1. **Variables additionally needed for primary analysis**

| **4.1 At least one variable defining “severe disease at presentation with FN”** | |
| --- | --- |
| **Variable** | **Attributes** |
| Severely reduced general condition at presentation | any binary coding (number or text) |
| Sepsis at presentation | any binary coding (number or text) |
| Triage category assigned by emergency team | Text or number |

1. **Variables additionally desired**

| **5.1 Priority 1** | |
| --- | --- |
| Location at fever detection (in the hospital / not in the hospital) | any binary coding (number or text) |
| Type of malignancy | Any format, e.g. diagnoses in text or numbers from chart.  If needed to generate manually, then best use our format:   1. ALL 2. AML 3. Hodgkin Lymphoma 4. Non-Hodgkin Lymphoma 5. CNS tumor 6. Other solid tumors |
| Bone marrow involvement at FN presentation | any binary coding (number or text) |
| Leucocyte count | Real number (_ _ . _ G/l) |
| Severe sepsis | Any binary coding (number or text) |
| **5.2 Priority 2** | |
| Time of first detection of fever | Date and Time (any format ) |
| Sex | any binary coding (number or text) |
| Age at FN diagnosis | Integer (_ _ years) or other format |
| Presence of any central venous access device at FN presentation | any binary coding (number or text) |
| Time from cancer diagnosis to FN | Integer (_ _ _ months) or other format |
| Max fever at FN diagnosis | Real number (_ _ . _°C) |
| Neutrophil count | Real number (_ _ . _ G/l) |
| Hemoglobin | Integer number (_ _ . _ g/l) or other format |
| Platelet count | Integer number (_ _ _ G/l) |
| Chemotherapy intensity (expected neutropenia) | 1. (none) 2. (≤10 days) 3. (>10 days) 4. (myeloablative)   Or other classification according to your center (then define, see 1.2) |
| Relapsed malignancy | any binary coding (number or text) |

**Appendix 2**

**
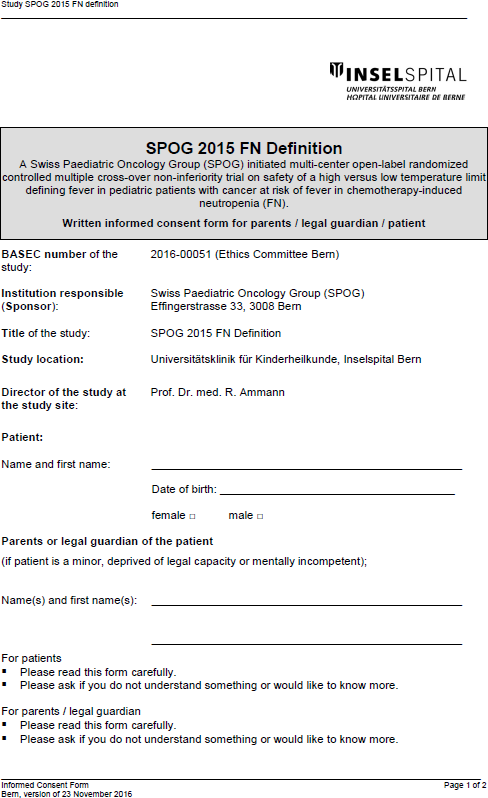
**


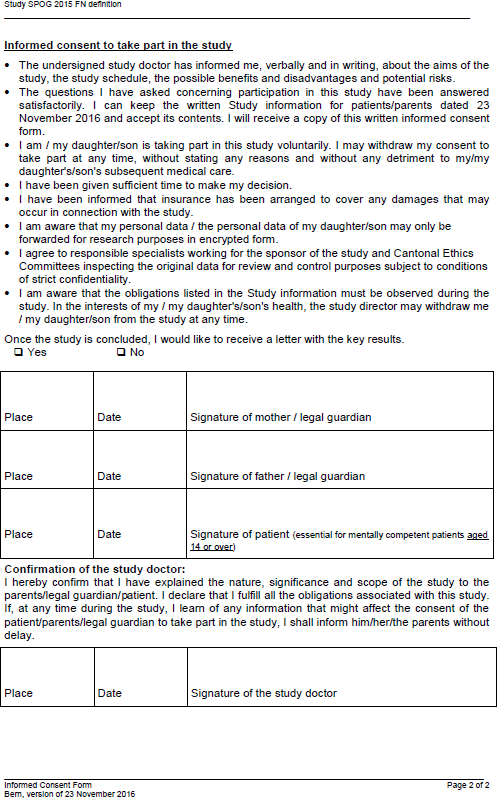


**Appendix 3**

**
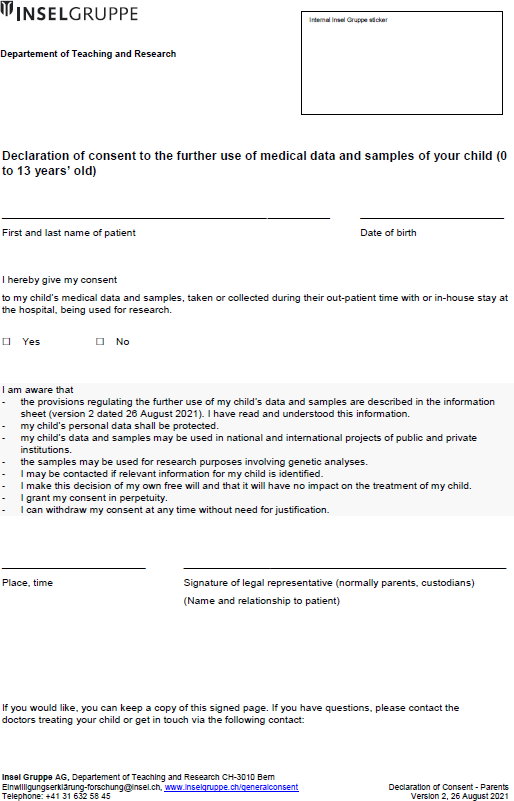
**


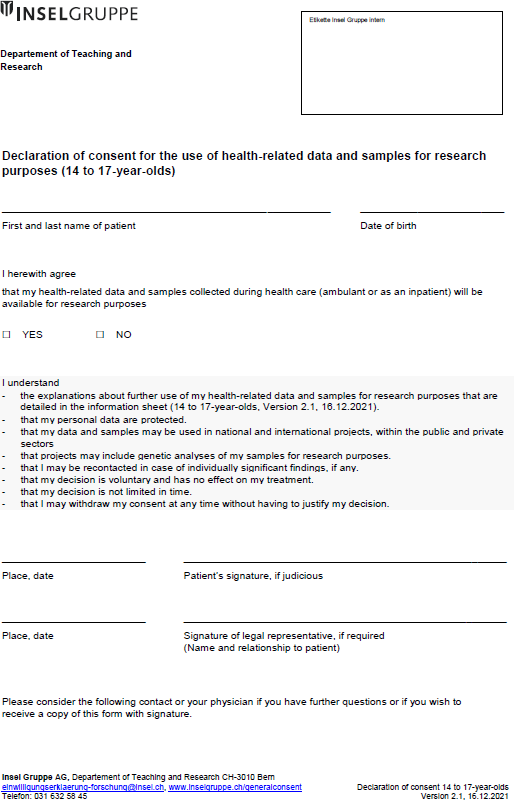


**Appendix 4**

**List of centers/countries agreeing to contribute data, as of 27.02.2023**

| **Country** | **Center/City** | **Approximate number of FN episodes** |
| --- | --- | --- |
| Australia | Various centers | 1900 episodes |
| Brazil | Various centers | 2000 episodes |
| Canada | Sick Kids / Toronto | not yet specified |
| Denmark | Copenhagen, possibly other Danish centers | 400 episodes |
| England | Various centers | 400 episodes |
| Germany | Frankfurt | 300 – 900 episodes |
| Greece | Athene Thessaloniki | not yet specified not yet specified |
| United States of America | Stanford Philadelphia | not yet specified not yet specified |
| Switzerland | 6 Centers from SPOG 2015 FND Study Bern outside of study | 360  150 |
